# Observation of Optical Precursor in Time‐Energy‐Entangled W Triphotons

**DOI:** 10.1002/advs.202501626

**Published:** 2025-05-08

**Authors:** Zhou Feng, Rui Zhuang, Sinong Liu, Guobin Liu, Kangkang Li, Yanpeng Zhang

**Affiliations:** ^1^ Key Laboratory for Physical Electronics and Devices of the Ministry of Education & Shaanxi Key Lab of Information Photonic Technique Xi'an Jiaotong University Xi'an 710049 China; ^2^ State Key Laboratory for Artificial Microstructure and Mesoscopic Physics and Frontiers Science Center for Nano‐optoelectronics School of Physics Peking University Beijing 100871 China

**Keywords:** optical precursor, six‐wave mixing, slow light effect, W‐state triphotons

## Abstract

In quantum communication and quantum computation, adding each entangled photon leads to an exponential increase in communication and computational capabilities. Utilizing a fifth‐order nonlinear process, narrow‐band non‐Gaussian W‐triphotons are experimentally generated through the spontaneous six ‐wave mixing (SSWM), but the manipulation of such entangled state in the group delay regime is still lacked. For the first time, the observation of optical precursors at the triphoton level is reported through the SSWM process. By appropriately adjusting the optical depth, optical precursors are effectively separated from the main wave through utilizing the slow‐light effect to manipulate the triphoton temporal correlations in the group delay regime. In biphoton and conditional two‐photon, the intensity ratio between the precursor and the main wave is tunable by adjusting the detuning and power of the laser. These pivotal breakthroughs mark a significant advancement in the study of optical precursors and open new possibilities for their application in quantum technologies.

## Graphical abstract

This study reports the observation of optical precursors at the triphoton level through the SSWM process. By appropriately adjusting the optical depth (OD), optical precursors are effectively separated from the main wave through utilizing the slow‐light effect to manipulate the triphoton temporal correlations in the group delay regime. These pivotal breakthroughs mark a significant advancement in the study of optical precursors.

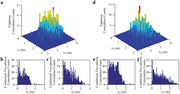

## 1 Introduction

Entangled multiphoton states are crucial for probing the foundations of quantum mechanics and are extensively utilized in optical quantum technologies.^[^ ^1^, ^2^, ^3^, ^4^, ^5^ ^]^ Especially for quantum communication and quantum computing, an increased photon number of entangled states significantly enhances information capacity and computation speed.^[^ ^6^, ^7^, ^8^, ^9^ ^]^ Based upon biphoton sources from spontaneous parametric downconversion (SPDC) and spontaneous four‐mixing (SFWM) processes, many efforts have developed multiphoton sources over the past thirty years by employing the different freedoms of photons, such as path, time multiplex, frequency multiplex and so on.^[^ ^10^, ^11^, ^12^ ^]^ However, efficiently creating dependable triphotons with nonclassical correlations distributed across all three particles has long been a goal for providing a quantum advantage. In the recent decade, several methods have developed for generating entangled triphoton states,^[^ ^13^, ^14^ ^]^ e.g., multiplexing of existing biphoton sources using linear optics combined with postselection techniques,^[^ ^4^, ^15^ ^]^ cascading two separated levels of SPDC/SFWM processes,^[^ ^16^, ^17^, ^18^, ^19^ ^]^ and other challenging attempts.^[^ ^9^, ^20^, ^21^, ^22^ ^]^ However, all these methods are difficult to offer a reliable and efficient triphoton source for research and applications due to dominant biphotons.

Directly using the single‐step process by a natural high‐order nonlinearity, without a doubt, is of great importance for developing a reliable triphoton source. Third‐order SPDC process,^[^ ^23^, ^24^, ^25^ ^]^ naturally converting one pump photon of higher energy into three daughter photons of low energy, is an ideal platform for generating triphotons but experimentally challenging owing to the lack of such a nonlinear optical material. Recently, a new method has been experimentally demonstrated to produce time‐energy‐entangled W‐class triphotons through spontaneous six‐wave mixing (SSWM) in coherent atomic media, achieving a reliable generation rate of ≈125 per minute.^[^ ^26^ ^]^ This groundbreaking achievement is conceptually novel and represents a significant advancement for the development of reliable and efficient genuine triphoton sources.

Unlike solid state systems, the atomic ensembles allow versatile narrowband biphoton/triphoton generation with advantageous properties,^[^ ^27^ ^]^ including long temporal coherence and controllable waveforms,^[^ ^28^ ^]^ which are ideal for applications like long‐distance quantum communications and information processing, bridging photons and neutral atoms.^[^ ^26^ ^]^ Specifically, the sharp optical response becomes a formidable tool for shaping waveforms and temporal correlations of entangled states:^[^ ^29^, ^30^ ^]^ the nonlinear susceptibility can precisely tune the coupling strength,^[^ ^31^, ^32^ ^]^ while the linear one mainly results in varying complex refractive index with the slow‐light effect of electromagnetically induced transparency (EIT).^[^ ^27^, ^33^, ^34^ ^]^ By precisely tuning the coupling strength in the nonlinear susceptibility,^[^ ^35^ ^]^ the quantum correlation of biphotons has been observed changing from the strong coupling characterized by the Rabi oscillation regime, to the slow‐light effect characterized by the group delay regime.^[^ ^36^, ^37^ ^]^

Optical precursor, referring to the propagation of the front of a step optical pulse in any dispersive medium, always traveling at the vacuum light velocity, was first theoretically demonstrated by Sommerfeld and Brillouin.^[^ ^38^, ^39^ ^]^ Experimentally, precursors have been predicted across various domains, including microwave,^[^ ^40^ ^]^ sound wave,^[^ ^41^, ^42^ ^]^ GaAs crystal,^[^ ^43^ ^]^ and water.^[^ ^44^ ^]^ Despite significant progress, the inability to isolate optical precursor waves from the main wave has hindered the broader acceptance of these findings.^[^ ^45^ ^]^ This limitation was addressed with the first successful separation of optical precursor waves from the main wave using a three‐energy‐level EIT atomic medium.^[^ ^39^, ^46^ ^]^ In recent decades, optical precursor propagation has been proven to exist at the single‐photon level.^[^ ^27^, ^47^, ^48^, ^49^ ^]^ In a cold atomic system, the sharp front‐edge spike of the biphoton waveforms is characterized as precursor propagation and separated by delaying the main wave packet of biphotons using the slow‐light effect in the group delay regime.^[^ ^36^, ^37^ ^]^ Subsequent studies have extended these investigations, deepened the understanding of the interaction between optical pulses and matter, and paved the way for potential applications of optical precursors in quantum communication.^[^ ^50^, ^51^, ^52^ ^]^ However, the study of precursor propagation at the triphoton level and the manipulation of its waveforms in the group delay regime is still lacking. Optical precursors at the triphoton level offer significant advantages for high‐fidelity signal transmission of entangled multiphoton states and are therefore crucial for advanced quantum information processing.^[^ ^53^, ^54^, ^55^, ^56^ ^]^

In this paper, we investigated the generation of time‐energy entangled biphoton and triphoton from a hot ^85^Rb vapor cell using SFWM and SSWM processes, respectively. In spontaneous multi‐wave mixing processes, energy conservation leads to the generated photons sharing strong spectral quantum correlations and implying strong correlations at the time of arrival of the photons, which results in an energy‐time entangled biphoton/triphoton state.^[^ ^33^, ^57^ ^]^ Notably, we present the first experimental observation of triphoton waveforms within the group delay regime, along with the first direct evidence of optical precursors at the triphoton level. By tracing one photon in the triphoton state away, partial entanglement still exists in the remaining bipartite subsystem, named the conditional two‐photon correlations, which conforms to the essential characteristics of the tripartite W class.^[^ ^9^ ^]^ In the waveforms of biphoton and conditional two‐photon correlations, the optical precursor can be precisely controlled and separated from the main wave by adjusting the detuning, power of the pump laser, as well as the optical depth of the system. These findings of optical precursors offer critical insights into the propagation dynamics of quantum light sources in dispersive media, and provide a foundational understanding of their influence on temporal correlations of biphoton/triphoton in the group delay regime, which are essential for potential quantum information processing applications.

## 2 Results

**Figure 1:**
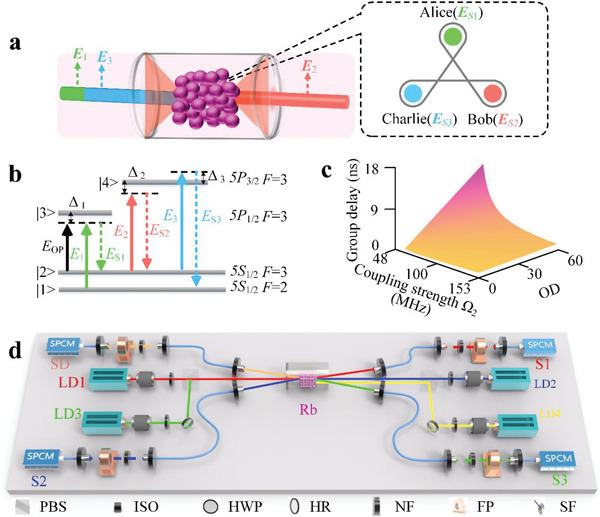
Generation of genuine time‐energy entangled triphotons directly via SSWM in a hot atomic vapor. a) Schematic of creating a triphoton state. b) The energy‐level diagram of ^85^Rb for the SSWM process. c) The group delay for *E*_S2_ as a function (*L*/*v_g_* − *L*/*c*) of varying coupling strength and optical depth (OD), with *P*_1_= 6 mW, Δ_1_= 2.0 GHz, Δ_2_= 0 MHz, Γ_41_= 2π*3 MHz and Γ_10_=Γ_41_ *0.01. d) Experimental setup. Three input beams *E*_1_, *E*_2,_ and *E*_3_ from three external cavity diode lasers (LD1‐LD3), are coupled into the center of the atomic vapor through isolators (ISO), half wave plates (HWP), high reflective mirrors (HR) and polarization beam splitters (PBS). Based on the SSWM process, the generated triphotons (*E*_*S*1_, *E*_*S*2_ and *E*_*S*3_) are coupled into single‐mode fibers (SF) with an angle near 4° to distinguish them from the incident laser beams, and then pass through optical narrowband filters (NF) and Fabry‐Perot cavity (FP) to filter out the possible biphotons noise. Finally, triphotons are detected by the single‐photon counting modules (SPCM) and recorded by a time‐to‐digit converter, where the maximum resolution time of our recording card is 813 fs.

The generation of genuine time‐energy‐entangled W‐class triphotons in one step can be realized via the SSWM of light‐atom interplay, as shown in **Figure** **1**a,b. In the presence of three counter‐propagating cw laser beams with tunable frequency detunings Δ_*j*_ and powers *P_j_* including a pump beam ($E_{1},\omega_{1},{\overset{⇀}{k}}_{1}$) and two coupling beams ($E_{2},\omega_{2},{\overset{⇀}{k}}_{2}$) and ($E_{3},\omega_{3},{\overset{⇀}{k}}_{3}$), backward‐propagating triphotons [Alice ($E_{S1},\omega_{S1},{\overset{⇀}{k}}_{S1}$), Bob ($E_{S2},\omega_{S2},{\overset{⇀}{k}}_{S2}$) and Charlie ($E_{S3},\omega_{S3},{\overset{⇀}{k}}_{S3}$)] are spontaneously emitted via Doppler‐broadened SSWM. Such an SSWM process occurs in a *L* = 7 cm long ^85^Rb vapor cell with a four‐level triple‐Λ atomic configuration, satisfying energy conservation (ω_1_ + ω_2_ +  ω_3_ = ω_*S*1_  + ω_*S*2_ + ω_*S*3_) and phase‐matching conditions (${\vec{k}}_{1}$ + ${\vec{k}}_{2}$ + ${\vec{k}}_{3}$   = ${\vec{k}}_{S1}$ *k*  + ${\vec{k}}_{S2}$ + ${\vec{k}}_{S3}$), resulting the strong spectral quantum correlat‐ions among triphoton states. Physically, the interaction Hamiltonian of the tiphoton generation process is defined as (neglecting the reflections from surfaces and employing the rotating‐wave approximation):^[^ ^26^, ^58^ ^]^

(1) $$H_{I}=\varepsilon_{0}\;\int_{V}d^{3}r\chi^{\left(5\right)}E_{1}^{\left(+\right)}E_{2}^{\left(+\right)}E_{3}^{\left(+\right)}E_{S3}^{\left(-\right)}E_{S2}^{\left(-\right)}E_{S1}^{\left(-\right)}+H.c$$

whereχ^(5)^ is the fifth‐order nonlinear susceptibility of SSWM process that can condition the triphoton generation rate and specifies the generation mechanism along with the photon intrinsic bandwidths, *V* is the volume illuminated by the input fields together, *H*.*C* is the Hermitian conjugate. The state vector of the triphotons can be derived using first‐order perturbation theory in the Schrödinger picture and can be formulated as follows:^[^ ^26^ ^]^

(2) $$\left|\psi \rangle \right.=\;\int \int \int \;d\omega_{S1}d\omega_{S2}d\omega_{S3}\chi^{\left(5\right)}\Phi \left(\frac{\Delta kL}{2}\right)\delta \left(\Delta \omega \right)\left|1\omega_{S1},1\omega_{S2},1\omega_{S3}\rangle \right.$$

Here, Δω  = ω_*S*1_  − ω_1_ + ω_*S*2_ − ω_2_ + ω_*S*3_ − ω_3_; Φ (Δ*kL*/2) =  sinc(Δ*kL*/2)*e* ^−*i*Δ*kL*/2^ is the phase‐mismatch longitudinal function that ascribes the three‐photon natural spectral width arising from their different group velocities; *L* is the interaction length;$\;\Delta k=\Delta \vec{k}\cdot \hat{z}$ is the phase mismatch; Dirac function (δ) is derived from the time integral in the steady‐state approximation, ensuring the energy conservation in the SSWM process.

To comprehensively understand the optical properties of the W‐type triphotons, we can examine their photon statistics through photon‐counting measurements. Consequently, we delve into the temporal correlation of triphotons by evaluating the Glauber third‐order correlation functions $G^{\left(3\right)}={|\left\langle 0|{\hat{a}}_{S1}^{†}{\hat{a}}_{S2}^{†}{\hat{a}}_{S3}^{†}|\psi \right\rangle |}^{2}$, ${\hat{a}}_{Si}^{†}$ is annihilation operator of *E_Si_*, which means the temporal waveforms of triphoton is jointly determined by the convolution of χ^(5)^ and Φ(Δ*kL*/2).^[^ ^18^, ^50^, ^51^ ^]^ As a consequence, we anticipate the appearance of two distinct regimes in three‐photon temporal correlation measurements, characterized by the damped Rabi oscillation regime dominated by χ^(5)^ and the group‐delay regime dominated by Φ(Δ*kL*/2).^[^ ^18^, ^52^, ^59^ ^,^ ^60^ ^]^ The three‐photon interference in damped Rabi oscillation regime has been observed in the previous work,^[^ ^26^ ^]^ but the manipulation of such entangled state in the group delay regime is still lacked.

Apart from the resonance linewidths governed by χ^(5)^, the temporal correlation of triphotons is also dependent on dispersion steming from the linear optical response. In our system, by the utilization of a weak input *E*_1_ beam coupled with an exceedingly large detuning Δ_1_ =   − 2 GHz from the transition |1〉 → |2〉. This outcome indicates that the group velocity of the *E*_*S*1_ photons closely approximates the speed of light in vacuum, *c*. Meanwhile, the coupling beams *E*_2_ and *E*_3_ operating under near‐resonant conditions, establish a “ʌ‐type” electromagnetically induced transparency configuration. In this setup, the coupling beam creates a transparency window for the photons *E*_*S*2_ and *E*_*S*3_, facilitating a slow‐light effect. Thus, the phase mismatch can be expressed as Δ*k*  = *k*_1_  + *k*_2_ + *k*_3_ − *k*_*S*1_ − *k*_*S*2_ − *k*_*S*3_ ≃ δ_2_/*V*_*g*2_ + *i*α_2_ + δ_3_/*V*_*g*3_ + *i*α_3_, where *V*_*g*2_ is the group velocity of photon *E*_*S*2_, *V*_*g*3_ is the group velocity of photon *E*_*S*3_, α_2_ and α_3_ are imaginary parts of Δκ and δ_*i*_ define the spectral distribution with respect to the central frequencies (ϖ_*Si*_) of the emitted *E_Si_* photon that is ω_*Si*_ = ϖ_*Si*_  + δ_*i*_. Consequently, the properties of triphotons temporal correlation in the group delay regime can be controlled by varying *V_gi_*. Figure 1c presents simulated results of the group delay time (*L*/*v*_*g*2_ − *L*/*c*) as a function of coupling strength Ω_2_ and the atomic density or OD. The optical depth is defined as *OD*  =  *N*σ_*ij*_ *L*, where σ_*ij*_ is the on‐resonance absorption cross section for the transition |i>→|j> and *N* is atomic density. The simulation reveals that the group delay time increases from 0 to 18 ns as the OD is improved, while it shows a negative correlation with the coupling strength. Therefore, the tiphoton waveforms can experimental control in the group delay regime by using the slow‐light effect. Figure 1d outlines the experimental setup for triphoton generation via the SSWM process. Detailed descriptions of the experimental configuration are provided in the Experimental Section.

### 2.1 Optical Precursor in Biphoton

To investigate optical precursor waves at the triphoton level, it is essential to first explore their behavior at the biphoton level, as this serves as a foundation for observing triphoton precursor waves more effectively. Thus, our initial focus lies in preparing biphoton precursors and separating them from the main wave. Biphotons (*E*_*S*1_ and *E*_*S*2_) are generated via the SFWM process by turning off laser LD3 (*E*_3_) and tuning the Fabry–Pérot cavity to transmit *E*_*S*2_ photons. The corresponding energy‐level diagram is presented in Figure S1 (Supporting Information). Under the FWM process, biphotons (*E*_*S*1_ and *E*_*S*2_) are spontaneously generated by absorbing one pump photon (*E*_1_) and one coupled photon (*E*_2_). This generation satisfies the phase‐matching condition (*k*_1_ +  *k*_2_ = *k*_*S*1_  + *k*_*S*2_) and energy conservation (ω_1_ +  ω_2_ = ω_*S*1_  + ω_*S*2,_ δ_1_ +  δ_2_ =  0).

**Figure 2:**
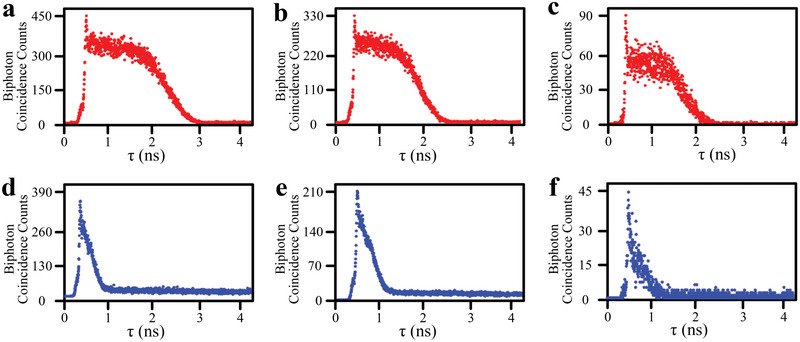
Two‐photon coincidence counting measurements. Collected about 1 min with a time bin of 0.25 ns for OD = 4.6. a) Input powers of the *E*_1_ and *E*_2_ are P_1_ =  6 mW and P_2_ =  4 mW, respectively. Δ_1_ is − 2.0 GHz and Δ_2_ is − 150 MHz. The raw biphoton generation rate is of 2.4e5 per second with the background accidentals of 60 per second. b) Δ_1_ changes to − 2.4 GHz. c) Δ_1_ changes to − 2.8 GHz. d) P_1_ is reduced to 4 mW and P_2_ is increased to 6 mW. e) P_1_ is reduced to 3 mW. f) P_1_ is reduced to 2 mW.

**Figure** **2** illustrates the temporal correlations of the biphoton state in the group delay regime via measuring the two photons coincidence counting, varying detuning (Δ_1_) and power (P_1_) of *E*_1_. In coincidence counting measurements, the detected *E*_*S*1_ and *E*_*S*2_ serve as the start triggering photon and stop triggering photon, respectively. τ is the relative time delay in the biphoton state, defined by τ  = *t*_*S*2_  − *t*_*S*1_ with *t*_*S*1/*S*2_ representing the triggering time of each detector. The waveforms of biphoton temporal correlations enveloped in Figure 2 demonstrate the distinct profiles in the group delay regime: attenuation gate‐like profile in Figure 2a–c and exponential decay profile in Figure 2d–f. Importantly, optical precursors are observed at the rising edge of the biphoton waveforms. It is worth to note that, at the single‐photon level, optical precursors are a relative concept, as their observation is inevitably influenced by dispersion and other overlapping optical responses. Theoretically, the biphoton amplitude *B*(τ) [in Equation S11, Supporting Information] contains two contributions in the frequency domain: the primary correlations of the biphoton [*B*_0_(τ)] and the Sommerfeld–Brillouin precursor component [*B_S_* (τ)]. In the group delay regime, the nonlinear coefficients (κ) in Equations S13 and S14 (Supporting Information) can be viewed as constants, and the photon generation process is primarily governed by the phase‐mismatch longitudinal function Φ(Δ*kL*/2)  =  sin*c*[Δ*kL*/2]*e ^*i*Δ*kL*/2^. In the SFWM, Δ*k* can be expressed as Δ*k*  = *k_s_*  − *k_as_* + *k*_1_ − *k*_2_ ≃ δ_2_/*V*_*g*2_ + *i*α_2_, where *V*_*g*2_ is group velocity of *E*_*S*2_ related to the linear susceptibility. The corresponding phase‐matched bandwidth is Δ ω_*g*2_ =  2π*V*_*g*2_/*L* ≃ |Ω_2_|^2^/(2Γ_41_OD) and the transmission EIT bandwidth is then expressed as Δω_*tr*2_ ≃ |Ω_2_|^2^/(2Γ_41_OD^1/2^). When the weak coupled input *E*_1_ with an large Δ_1_ and *E*_2_ with an weak power, the temporal correlations of biphoton are manipulated by slow‐light effect and small imaginary part of Δ*k*, resulting attenuation gate‐like profile with $e^{-\alpha_{2}L}\ll 1$ in Figure 2a–c. From Figure 2a–c, the intensity ratio of the precursor to the main wave increases with larger detuning. Following with increased P_2_, the waveforms of biphoton in Figure 2d–f are shaped as exponential decay profile. From Figure 2d–f, the intensity ratio of the precursor to the main wave also increases with decreased P_1_. These results present the optical precursors and slow‐light properties of biphotons temporal correlation in the group delay regime. Moreover, to demonstrate the nonclassicality nature of the biphoton, we examine the violation of the Cauchy‐Schwarz inequality (Equation S15, Supporting Information).^[^ ^61^, ^62^ ^]^ The violation of the Cauchy–Schwarz inequality in biphoton reaches factors of 10 − 40 in Figure 2.

### 2.2 Optical Precursor in Triphoton

Based on above time‐energy entangled biphotons, genuine time–energy‐entangled W‐class triphotons can be realized via the SSWM by turning on laser LD3 (*E*_3_) presented in Figure 1d.

**Figure 3:**
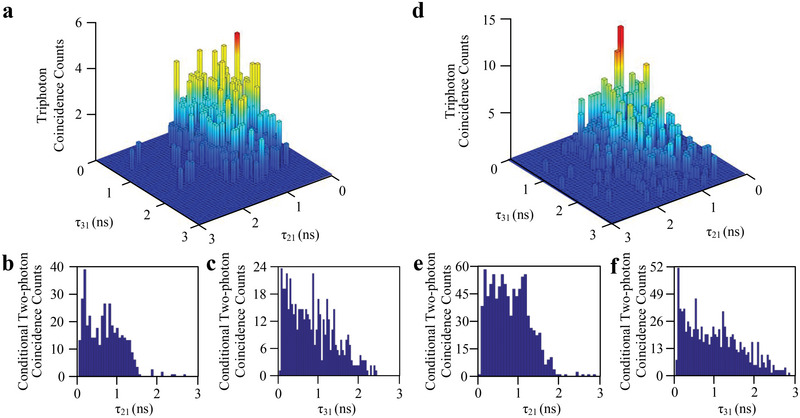
Triphoton coincidence counting measurements. a) Triphotons are collected in 1 h with a time bin of 0.25 ns at an optical depth of 1.04. The raw tiphoton generation rate is of 62 min^−1^ with the background accidentals of 2 min^−1^. The power of the input beams *E*_1_, *E*_2_ and *E*_3_ are 5, 7, and 9 mW, respectively. Δ_1_ is − 2.0 GHz, Δ_2_ is − 150 MHz and Δ_3_ is 50 MHz. b,c) Conditional two‐photon coincidence counts as the function of τ_21_ and τ_31_ in (a) by tracing the third photon *E*_*S*2_ and *E*_*S*3_, respectively. d) Triphoton coincidence counts collected for OD = 63.76, and all other conditions remained identical to those in (a). The raw tiphoton generation rate is of 105 min^−1^ with the background accidentals of 3 min^−1^. e,f) conditional two‐photon coincidence counts as the function of τ_21_ and τ_31_ in (d) by tracing the third photon *E*_*S*2_ and *E*_*S*3_, respectively.

Within in time window of 195 ns, the detection of an *E*_*S*1_‐photon as triggered the start of a coincidence event that ended with the detection of subsequent *E*_*S*2_‐ and *E*_*S*3_‐photons. The coincidence counting measurements of triphoton display as the histogram in the parameter space (τ_21_,τ_31_) in **Figure** **3**, where τ_21_ = τ_2_  − τ_1_ and τ_31_ = τ_3_  − τ_1_ are respectively the relative time delays with τ_*j*_ being the triggering time of the SPCM*_j_*.

According to the linear susceptibilities in SSWM, the phase‐matched bandwidths can be calculated as Δω_*g*2_ ≃ (|Ω_2_|^2^ + Γ_42_Γ_22_)/(2Γ_42_OD) and Δω_*g*3_ ≃ (|Ω_3_|^2^ + Γ_41_Γ_11_)/(2Γ_41_OD). Theoretically, when Δω_*gi*_ is smaller than both the effective Rabi frequency (Ω_e_) and effective dephasing rate (Γ_e_), the temporal correlations of triphoton are mainly decided by the phase‐mismatch longitudinal function (detials in the Supporting Information).^[^ ^31^ ^]^ In this case, the triphoton amplitude *B*(τ) in Equation S26 (Supporting Information) comprises the primary component *B*_0_(τ) in Equation S28 (Supporting Information) and the Sommerfeld–Brillouin precursor component *B_S_* (τ) in Equation S29 (Supporting Information). As shown in Figure 3a, the waveform of triphoton temporal correlations exhibits a gate‐like profile, confirming that the triphoton resides in the group delay regime dominated by Φ(Δ*kL*/2) with slow‐light effect. By such precisely controlling, a pronounced leading edge in the triphoton waveform is observed near τ_21_ = τ_31_  =  0, with counts ≈1.5 times greater than the main waveform. This leading edge can be viewed as the optical precursor wave at the triphoton level, reported for the first time. To verify this feature more clearly and confirm the W class of triphoton state, tracing out the arrival time of one photon from each triphoton event, we analyzed the correlation properties of bipartite subsystems by integrating coincidence counts.^[^ ^26^ ^]^ The temporal waveforms of conditional two‐photon along in τ_21_ (*E*_*S*1_ and *E*_*S*2_) and τ_31_ (*E*_*S*1_ and *E*_*S*3_) as shown in Figure 3b,c, respectively, in which, the optical precursor waves are visible and separated at their rising edge. The persistence of optical precursor waves in conditional two‐photon waveforms strongly supports the presence of quantum properties characteristic of a W state. To demonstrate the nonclassicality nature of the W triphoton state, we examine the violation of the Cauchy‐Schwarz inequality.^[^ ^61^, ^62^ ^]^ By normalizing the threefold coincidence events to the flat background counts along with the additional autocorrelation measurement of the collected *E*_*S*1_, *E*_*S*2_ and *E*_*S*3_ photons, we obtain a clear violation of the Cauchy–Schwarz inequality (Equation S31, Supporting Information) by a factor of 12.6 in Figure 3a with the normalized third‐order correlation function value of 9.

The observation of optical precursor can be precisely controlled by increasing the OD, thereby enhancing the slow‐light effect. In Figure 3d, the waveform of triphoton temporal correlations is observed by changing OD to 63.76 with the 120 °C temperature of the Rb vapor cell, where a distinct sharp leading edge in the waveform signifies the presence of the triphoton precursor. Here, the violation of the Cauchy–Schwarz inequality reaches a factor of 6.8 with the normalized third‐order correlation function value of 6.6. As the OD increases from 1.04 to 63.76, the phase‐matched bandwidths Δω_*g*2_ and Δω_*g*3_ become significantly smaller compared to Figure 3a. The histogram in Figure 3e,f illustrates the conditional two‐photon coincidence counts in τ_21_ and τ_31_, respectively, in which, the the main waves are delayed with the enhanced slow‐light effect.

### 2.3 Optical Precursor in Conditional Two‐Photon Correlations

**Figure 4:**
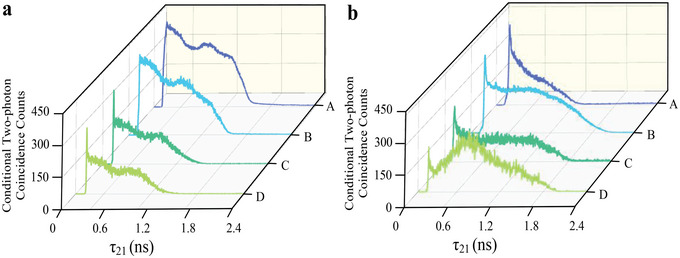
Conditional two‐photon coincidence counting measurements along τ_21_. Collected over 1 h with a time‐bin width of 0.25 ns. a) The input powers of the *E*_1_ and *E*_3_ are 6 and 9 mW, respectively. Δ_1_ is − 2.0 GHz, Δ_2_ is − 150 MHz and Δ_3_ is 50 MHz. The temperature of the ^85^Rb vapor cell is maintained at 70 °C. The power of *E*_2_ is varied (A: 11 mW, B: 9 mW, C: 7 mW, and D: 5 mW). b) The input powers of *E*_1_, *E*_2_ and *E*_3_ are 6 mw, 11 mw and 9 mw, respectively. The temperature of the ^85^Rb vapor cell is varied (A: 60 °C, B: 70 °C, C: 80 °C, and D: 90 °C).

For further investigate the optical precursor by W‐class triphotons, we manipulate the condional two‐photon correlations along τ_21_ in **Figure** **4**. In such a state, by tracing out *E*_*S*3_ photon before triphotons reconstructing process leaves the remaining two *E*_*S*1_ and *E*_*S*2_ still strongly entangled (see Supporting Information for details). Although there have some remanent and unexpected biphoton coincidence noise, this contributes the background of the conditional two‐photon waveforms that can be efficiently eliminated by triphotons reconstructing process.^[^ ^26^ ^]^ Here, a clear temporal separation between the precursor wave and the main wave is observed by adjusting the power of the input laser, as well as the optical depth of the system. In Figure 4a, the temperature of Rb vapor cell is maintained at 70 °C with the power of *E*_1_ and *E*_3_ are 6 and 9 mW, respectively, while the power of *E*_2_ is varied from trace A to trace D. As the power of *E*_2_ decreases from 11 to 5 mW, a distinct precursor wave becomes increasingly visible at the leading edge of the conditional two‐photon coincidence counts. This enhancement is attributed to the dual role of in *E*_2_, enabling *E*_*S*2_ photon propagation via EIT and modulating group velocity through the slow‐light effect.^[^ ^63^ ^]^ In the SSWM process, the optical precursor and main waves exhibit different sensitivities to the coupling field. The main wave shows significantly dependence on the dispersion of medium and delayed response. These properties fundamentally differentiate the generation and propagation of the optical precursor from that of the main wave.^[^ ^27^, ^48^ ^]^

In Figure 4b, the temperatures of Rb vapor cell are varied from 60 to 90 °C in the traces A‐D, corresponding OD is changing from 1.04 to 9.78. It is observed that the precursor appears at the leading edge of the conditional two‐photon coincidence counts, exhibiting negligible relative delay during propagation through the medium. Furthermore, the precursor progressively separates from the main wave packet as the OD increases. For the precursor to be distinctly observed, the OD must be sufficiently large. However, the delayed group velocity in the medium decreases the production efficiency of conditional two‐photons. These results of optical precursors provide a foundational understanding of propagation dynamics of temporal correlations of W‐class triphoton in the group delay regime.

## 3 Conclusion

In summary, we present the first experimental observation of optical precursors at the triphoton level by manipulating triphoton waveforms within the group delay regime via the SSWM process in atomic ensembles. Furthermore, to verify the characteristics of the triphoton W class, the optical precursors are precisely controlled and separated from the main wave in conditional two‐photon correlations through the slow‐light effect. Benefiting from the fine‐tuning of the light‐matter coupling between the atomic states and external optical fields by system multi‐parameters, the produced quantum correlations of triphoton are therefore demonstrated could be in either the Rabi oscillation regime or the group delay regime.^[^ ^26^ ^]^ The observation of these unique properties, such as three‐photon interference and optical precursors, is significant for providing a reliable and efficient genuine triphoton source. These findings represent a significant advancement in the study of optical precursors on the propagation dynamics of entangled multiphoton states, offering a promising pathway for quantum information processing.

## 4 Experimental Section

The experimental configuration for the generation of biphoton and triphoton precursors is illustrated in Figure 1. Photons are produced in a paraffin‐coated ^85^Rb vapor cell with a length of *L* = 7 cm. Three continuous‐wave beams, *E*_1_, *E*_2_ and *E*_3_, were generated by external cavity diode lasers LD_1_, LD_2_ and LD_3_ respectively. The pump field *E*_1_ (frequency ω_1_, wave vector *k*_1_, Rabi frequency Ω_1_, horizontal polarization) was employed with a wavelength *λ*_1_ = 795 nm and large detuned from the resonance transition between level |1〉 and level |3〉, as shown in Figure 1b. Coupling fields *E*_2_ (*λ*_2_ = 780 nm, ω_2_, *k*_2_, Ω_2_) and *E*_3_ (*λ*_3_ = 780 nm, ω_3_, *k*_3_, Ω_3_) are vertical polarization, couple |2〉 → |4〉 and |1〉 → |4〉, respectively. Triphoton *E*_*S*1_, *E*_*S*2_ and *E*_*S*3_ were spontaneously generated with satisfying phase‐matching conditions (*k*_1_ + *k*_2_ +  *k*_3_ = *k*_*S*1_  + *k*_*S*2_ + *k*_*S*3_) and energy conservation (ω_1_ + ω_2_ +  ω_3_ = ω_*S*1_  + ω_*S*2_ + ω_*S*3_, δ_1_ + δ_2_ +  δ_3_ =  0). δ_*i*_ (i = 1, 2 and·3) is the spectral distribution with respect to the central frequencies (ϖ_*Si*_) of the emitted *E_Si_* photon that is ω_*Si*_ = ϖ_*Si*_  + δ_*i*_. Consequently, this process allows to acquire time–energy‐entangled W‐state triphotons (Alice, Bob and Charlie), as depicted in Figure 1a, illustrating their relationship. To filter out un‐correlated photons and other photons, Fabry–Pérot cavities (with a bandwidth ≈600 MHz) and band pass filters (F) were utilized. The bandwidth, transmission efficiency, and extinction ratio of the used filters are 650 MHz, 80%, and 60 dB, respectively. To further reduce the occurrence of false trigger events caused by double pairs, an additional SPCMd synchronized with SPCM3 was introduced that acts as a diagnosis detector in conjunction with SPCM1 and SPCM2.^[^ ^26^ ^]^ It is noticed that, in photon joint clicks, a significant portion of accidental photons can be eliminated; however, some persist, including biphotons generated from the FWM process and dark counts of the SPCMs.^[^ ^26^ ^]^

The pump beam *E*_1_ operates at a lower power and a far‐off resonant wavelength, which can effectively suppress quantum atomic noise and ensure atomic population remains in the ground state.^[^ ^64^ ^]^ Meanwhile, the coupling beams *E*_2_ and *E*_3_ operating under near‐resonant conditions, establish a “ʌ‐type” electromagnetically induced transparency configuration. In this setup, the coupling beam creates a transparency window for the photons *E*_*S*2_ and *E*_*S*3_, facilitating a slow‐light effect. Additionally, a strong on‐resonance optical‐pumping field was applied from LD4 (*E_OP_*, ω_*OP*_, *λ =* 795 nm) on the transition |2〉 → |3〉, which effectively eliminates residual atoms in level |2〉 and mitigates the noise from the Raman scattering. In order to detect the entangled triphoton, a Time‐to‐Digital Conversion (TDC) technique was employed, with the relevant details illustrated in Figure S9 (Supporting Information). By disabling SPCM3 and removing the delay component of ***E***_*S*1_, conditional two‐photon counts were obtained. Additionally, biphotons could be generated by switching off LD3 and setting experimental parameters in the FWM process.

To analyze the optical properties of the triphotons generated from a four‐level system, it is crucial to examine the triphoton coincidence counting measurements. Moreover, considering that the narrow bandwidths of the generated triphotons (less than GHz) are comparable to or smaller than the spectral resolution of the single‐photon detectors used in the experiment. The averaged triphoton coincidence counting, assuming ideal detection efficiency, is defined as follows:^[^ ^26^, ^65^ ^]^

(3) $$R_{cc}={\left|\left\langle 0|E_{S3}^{\left(+\right)}\left(\tau_{3}\right)E_{S2}^{\left(+\right)}\left(\tau_{2}\right)E_{S1}^{\left(+\right)}\left(\tau_{1}\right)|\Psi \right\rangle \right|}^{2}={\left|B\left(\tau_{21},\tau_{31}\right)\right|}^{2}$$

where τ_21_ = *t*_*S*2_  − *t*_*S*1_ and τ_31_ = *t*_*S*3_  − *t*_*S*1_. τ_31_ = *t*_*S*3_  − *t*_*S*1_, *t_Si_* = *r_Si_*/*c* , with *r_Si_*_i_ representing the optical path of the photon from the producing surface of the SSWM to the detector. *B*(τ_21_,τ_31_) is triphoton amplitude.

## Conflict of Interest

The authors declare no conflict of interest.

## Supporting information



Supporting Information

## Data Availability

The data that support the findings of this study are available in the supplementary material of this article.
