# Supplementary material for: Observation of Optical Precursor in Time‐Energy‐Entangled W Triphotons
Source: Adv Sci (Weinh). 2025 May 8;12(27):2501626. doi: 10.1002/advs.202501626 (PMC12279198; doi:10.1002/advs.202501626)
Supplement: Supplementary file 1 — Supporting Information [file ADVS-12-2501626-s001.docx]

Observation of Optical Precursor in Time-energy-entangled W Triphotons

Zhou Feng1, Rui Zhuang1*, Sinong Liu1, Guobin Liu1, Kangkang Li2* and Yanpeng Zhang1*

1. Key Laboratory for Physical Electronics and Devices of the Ministry of Education & Shaanxi Key Lab of Information Photonic Technique, Xi’an Jiaotong University, Xi’an 710049, China

2. State Key Laboratory for Artificial Microstructure and Mesoscopic Physics and Frontiers Science Center for Nano-optoelectronics, School of Physics, Peking University, 100871 Beijing, China

E-mail: zhuangray@stu.xjtu.edu.cn, kangkangli@pku.edu.cn, ypzhang@mail.xjtu.edu.cn

**1. Theory of Time-Energy-Entangled Biphoton Generation in an Atomic Vapor**

**2. Theory of Time-Energy-Entangled W Triphoton Generation in an Atomic Vapor**

**3. Theory of Time-Energy-Entangled Conditional Two-photon Generation in an Atomic Vapor**

**4. Simulation of Precursor in Triphoton**

**5. Procedure for Reconstructing Triphoton Coincidence Counts**

**1. Theory of Time-Energy-Entangled Biphoton Generation in an Atomic Vapor**

To calculate the resultant two-photon state stemming from the four-wave mixing (FWM) process at the output surface of the medium, we use the effective interaction Hamiltonian to calculate the two-order correlation function $G^{(2)}$ of the generated photons. The Hamiltonian for the four-wave mixing process can be written as (neglecting reflections from surfaces and employing the rotating-wave approximation):

$H_{I}=\varepsilon_{0}\int_{V} d^{2}r\chi^{(3)}E_{1}^{(+)}E_{2}^{(+)}E_{S2}^{(-)}E_{S1}^{(-)}+H.c$ (S1)

where $\chi^{(3)}$ is the third-order nonlinear susceptibility of the FWM process, $H.c$ is the Hermitian conjugate, $E_{i}^{\left( + \right)}$ represents the positive-frequency part of the input beams. Here, the generated $E_{S1}$ and $E_{S2}$ photons are described by the quantized electric fields,

$E_{Sj}^{(-)}=\sum_{k_{Sj}} E_{Sj}a_{j}e^{i\left( k_{Sj}z-\omega_{Sj}t \right)}$, (S2)

where $a_{j}$ symbolizes the annihilation operator for the mode with the wavenumber $k_{Sj}$ and angular frequency $\omega_{Sj}$. Additionally, $E_{Sj}=i\sqrt{\hbar\omega_{Sj}/2\varepsilon_{0}n_{Sj}^{2}L}$. On the other hand, three input continuous-wave (cw) lasers $E_{1}$ and $E_{2}$ are taken as classical plane waves,

$E_{1}^{(+)}=E_{1}e^{i\left( k_{1}z-\omega_{1}t \right)}$ and $E_{2}^{(+)}=E_{2}e^{i\left( {-k}_{2}z-\omega_{2}t \right)}$ (S3)

Substituting the electric field of the input and output into Equation (S1) we can get:

${\overset{\wedge}{H}}_{I}=W_{1}\int d\omega_{S1}d\omega_{S2}\kappa\Phi(\Delta kL/2)\hat{a}_{S1}^{\dagger}\hat{a}_{S2}^{\dagger}e^{-i\Delta\omega t}+H.c.$ (S4)

where $\kappa\left( \omega_{i} \right)=-i\sqrt{\varpi_{S1}\varpi_{S2}/c^{2}}\chi^{(3)}(\omega_{S1},\omega_{S2})E_{1}E_{2}$ is the nonlinear parametric coupling coefficient; $\hat{a}_{S1}^{\dagger}$ and $\hat{a}_{S2}^{\dagger}$ represent the photon generation operators for the output modes $E_{S1}$ and $E_{S2}$, respectively. $\Phi(\Delta kL/2)=\sin c(\Delta kL/2)*e^{i\Delta kL/2}$ is the longitudinal detuning function that determines the natural spectral width; $W_{1}$ is a constant; *L* is the length of the rubidium atomic vapor cell; $\Delta k=k_{s}-k_{as}+k_{1}-k_{2}$ is the phase mismatching.

The state vector of the biphotons can then be derived using first-order perturbation theory,^[1-2]^ which is

$\left| \psi\right\rangle=\frac{-i}{\hbar}\int_{-\infty}^{+\infty} dt\overset{\wedge}{H_{I}}\left| 0 \right\rangle$ (S5)

Based on the work in ^[3]^ and ignoring the vacuum term that has no effect in photon clicks, the biphoton state can be formulated as:

$\left| \psi\right\rangle=\sum_{k_{S1}} \sum_{k_{S2}} F\left( k_{S1},k_{S2} \right)a_{k_{S1}}^{\dagger}a_{k_{S2}}^{\dagger}\left| 0 \right\rangle$ (S6)

where the biphoton spectral function $F\left( k_{S1},k_{S2} \right)$ is defined as

$F\left( k_{S1},k_{S2} \right)=A\chi^{(3)}\Phi\left( \Delta kL/2 \right)\delta\left( \omega_{1}+\omega_{2}-\omega_{S1}-\omega_{S2} \right)$ (S7)

with *A* being a grouped constant. Dirac function ($\delta$) comes from the time integral in the steady-state approximation, ensuring the energy conservation in the SFWM process. From the perspective of atomic population, this energy conservation implies that after a biphoton generation cycle, the population returns to its initial ground state |1⟩.

**Figure S1.** a) Energy-level diagram of hot ^85^Rb atoms for biphoton generation. The light-atom interaction occurs in a four-level triple-Λ-type atomic configuration, where the two ground states are denoted by $\left. \left| 1 \right. \right\rangle$ and $\left. \left| 2 \right. \right\rangle$, and the two excited states by $\left. \left| 3 \right. \right\rangle$ and$\left. \left| 4 \right. \right\rangle$. Initially, all atomic population is prepared at $\left. \left| 1 \right. \right\rangle$. To ensure no residual atomic population is distributed in $|\left. 2 \right\rangle$, an additional optical pumping beam $E_{OP}$ is applied resonantly with the atomic transition $\left. |2 \right\rangle⟷\left. |3 \right\rangle$. A weak pump laser $E_{1}$ is applied to $\left. |1 \right\rangle\to\left. |3 \right\rangle$ with a large, fixed red frequency detuning $\Delta_{1}$, $E_{2}$ applied to atomic transition $\left. |2 \right\rangle\to\left. |4 \right\rangle$ with frequency detuning $\Delta_{2}$. b) The intensities of both the transmission bandwidth (black line) and the phase-matched bandwidth (red line) in the SFWM process vary with the power of $E_{2}$, with $\Gamma_{41}$=2π*3 MHz.

According to the energy level in **Figure** S1, the perturbation chain of generated photons in the FWM process can be expressed as:^[4-5]^ $\rho_{11}^{(0)}\underset{\to}{\omega_{1}}\rho_{31}^{(1)}\underset{\to}{\omega_{S1}}\rho_{21}^{(2)}\underset{\to}{\omega_{2}}\rho_{41}^{(3)}$. Here, $\omega_{i}$ and $\omega_{Si}$ are the frequency of input laser and output photons, respectively. In the dressing dressed state picture, by solving the density-matrix equations and considering a one-dimensional Doppler effect, third-order nonlinear susceptibility can be written as:

$\chi_{S2}^{(3)}=\frac{1}{\varepsilon_{0}\hbar^{3}}\frac{2N\mu_{13}\mu_{24}\mu_{23}\mu_{14}}{\left[ \left( i\Gamma_{31}+\Delta_{1} \right)\left( i{\Gamma_{e}}_{1}+W_{D+}\delta_{2}+\frac{{\Omega_{e}}_{2}}{2} \right)\left( i{\Gamma_{e}}_{2}+W_{D+}\delta_{2}+\frac{\Delta_{2}}{2}-\frac{{\Omega_{e}}_{2}}{2} \right) \right]}$ (S8)

where $\Omega_{e2}=\left( \Delta_{1}^{2}+4\Omega_{2}^{2}+4\Gamma_{21}\Gamma_{41} \right)^{1/2}$ is the effective resonance Rabi frequencies; $\Gamma_{e2}=\left( \Gamma_{21}+\Gamma_{41} \right)/2$ is the effective resonance linewidth, which is inversely proportional to the correlation time of biphoton temporal correlation. $\Gamma_{ij}=\left( \Gamma_{i}+\Gamma_{j} \right)/2$ is the decoherence rate between $\left. |i \right\rangle$ and $\left. |j \right\rangle$; $\Delta_{i}=\omega_{i}-\omega_{j}$ is detuning defined as the difference between the resonant transition frequency $\omega_{i}$ and laser frequency $\omega_{j}$ of $E_{i}$; $\Omega_{i}={\mu_{ij}E_{i}}/\hbar$ is the Rabi frequency, $\delta_{i}$ represents the deviations around the corresponding central frequency $\varpi_{Si}$ of generated photons that is $\omega_{Si}=\varpi_{Si}+\delta_{i}$ (*i*=1, 2), where $\left| \delta_{i} \right|\ll\varpi_{Si}$. Further, the frequency entanglement also can satisfy $\delta_{1}+\delta_{2}=0$. $W_{D-}$ and $W_{D+}$ are Doppler broadening factor, and $w_{D-}=1-v/c$, $w_{D+}=1+v/c$, respectively.

Meantime, we find that the linear susceptibility of the $E_{S1}$ is approximately zero ($\chi_{S1}\approx0$), while the linear susceptibility of the $E_{S2}$ field can be represented as:

$\chi_{S2}=\int f\left( v \right)dv\frac{N\mu_{41}^{2}}{\varepsilon_{0}\hbar}\frac{-1}{\left[ W_{D+}\delta_{2}+\Delta_{2}-i\Gamma_{41}-\frac{\left| \Omega_{2} \right|^{2}}{W_{D+}\delta_{2}-i\Gamma_{21}} \right]}$ (S9)

Generally, the shape of the biphoton correlation wave function is determined by both the nonlinear parametric coupling coefficient ($\kappa(\omega)$) and the longitudinal detuning function ($\Phi(\Delta kL/2)$). And, they are affected interplay of four key parameters: the effective resonance Rabi frequencies ($\Omega_{ei}$), the resonance linewidth ($\Gamma_{ei}$), the transmission (EIT) bandwidth ($\Delta\omega_{tri}=\sqrt{\frac{c}{\omega_{41}L}\frac{-\left( \Gamma_{10}\Gamma_{41}+\left| \Omega_{2} \right|^{2} \right)^{4}}{2\Gamma_{41}\left( \Gamma_{10}\Gamma_{41}+\left| \Omega_{2} \right|^{2} \right)^{2}+2\left( \Gamma_{41}{\Gamma_{10}}^{2}+\Gamma_{10}\left| \Omega_{2} \right|^{2} \right)\left( \Gamma_{10}+\Gamma_{41}-2\left( \Gamma_{10}\Gamma_{41}+\left| \Omega_{2} \right|^{2} \right) \right)}}$) and the phase-matched bandwidth ($\Delta\omega_{gi}=2\pi V_{g}/L=\left( \frac{8\pi^{2}}{OD\Gamma_{41}} \right)\left( \frac{\left( \Gamma_{10}\Gamma_{41}+\left| \Omega_{2} \right|^{2} \right)^{2}}{\left| \Omega_{2} \right|^{2}-{\Gamma_{10}}^{2}} \right)$).^[6]^

Figure S1b illustrates the variation in the intensities of both the transmission bandwidth (black line) $\Delta\omega_{tri}$ and the phase-matched bandwidth (red line) $\Delta\omega_{gi}$ in the SFWM process as a function of the power of$E_{2}$. It is evident that when the power of $E_{2}$ is below 4.45 mW, the intensity of the $\Delta\omega_{gi}$ exceeds that of the $\Delta\omega_{tri}$. According to Reference ^[6]^, this results in a coincidence count profile with an attenuation gate profile. However, when the power of $E_{2}$ exceeds 4.45 mW, this relationship is reversed and corresponding coincidence count profile becomes into gate profile. This result is consistent with the results in the paper.

**Figure S2.** The group delay for $E_{S2}$ as a function ($L/{v_{g}}\boldsymbol{-}L/c$) of detuning ($\Delta\omega_{2}$), with OD=4.6, $P_{1}$= 6 mW, $\Delta_{1}$=2.0 GHz, $\Delta_{2}$=150 MHz,$\Gamma_{31}$=2π*3 MHz, $\Gamma_{21}$=$\Gamma_{31}$*0.01. a) $P_{2}$=8 mW, b) $P_{2}$=9 mW, c) $P_{2}$=10 mW and d) $P_{2}$=11 mW.

To analyze the optical properties of the generated biphoton from a four-level system, we need to consider the biphoton coincidence counting measurement. Moreover, considering that the narrow bandwidths of the generated biphotons (less than GHz) are comparable to or smaller than the spectral resolution of the single-photon detectors used in our experiment. Assuming perfect detection efficiency, the averaged biphoton coincidence counting rate is defined by ^[6]^

$R_{cc}=\left| \left\langle0 | E_{S2}^{\left( + \right)}(\tau_{2})E_{S1}^{\left( + \right)}(\tau_{1}) | \Psi\right\rangle\right|^{2}=\left| G^{(2)} \right|^{2}$ (S10)

$G^{(2)}$ is the second-order intensity correlation function of biphoton, which can be written as:

$G^{(2)}=\left| B\left( \tau\right) \right|^{2}$ (S11)

where $\tau=t_{S2}-t_{S1}$. $t_{Si}={r_{Si}}/c$, $r_{Si}$ is the optical path of photon from the producing surface of the FWM to the detector. The biphoton amplitude ($B(\tau)$) comprises two components that originate from different regions in frequency space:

$B\left( \tau\right)=B_{0}\left( \tau\right)+B_{s}\left( \tau\right)$ (S12)

In the function, $B_{0}\left( \tau\right)$ represents the primary contribution of the biphoton, encompassing the majority of its energy, and is derived from a numerical integration over the central region (Region I) in **Figure** S2a that extends $\Delta\omega$ from ${-\Omega}_{c}/2$ to $\Omega_{c}/2$, which can be written as:^[7]^

$B_{0}\left( \tau\right)=W_{2}\int_{-\Omega_{c}/2}^{\Omega_{c}/2} \kappa\left( \omega\right)\Phi(\Delta kL/2)e^{-i\left( \omega\tau\right)}d\omega$ (S13)

$B_{s}\left( \tau\right)$ is the Sommerfeld–Brillouin precursor part of biphoton and is obtained by integrating over region II in Figure S2a. For an oscillatory integral of the form $f=\int_{-\infty}^{\infty} F\left( \omega\right)e^{i\theta\left( \omega\right)}d\omega$, where $F\left( \omega\right)$ varies slowly as compared to the real phase $\theta\left( \omega\right)$, the dominant contribution to the integral occurs when the derivative $\theta^{'}\left( \omega_{d} \right)$is equal to zero.^[7]^ With the contribution of the end points neglected, this integral is $f_{d}=\sqrt{2\pi i/\theta^{''}\left( \omega_{d} \right)}F\left( \omega_{d} \right)e^{i\theta\left( \omega_{d} \right)}.$^[8]^ When there are several points of stationary phase, it follows that the Sommerfeld–Brillouin portion of the wave packet is:^[7]^

$B_{S}\left( \tau\right)=\sum_{\omega_{d}} \frac{\kappa\left( \omega_{d} \right)\Phi(\Delta kL/2)}{\sqrt{-i2\pi\theta^{''}\left( \omega_{d} \right)}}e^{-i\left( \omega_{d}\tau\right)}$ (S14)

Where $\kappa\left( \omega\right)=-i\sqrt{\varpi_{S1}\varpi_{S2}/c^{2}}\chi^{(3)}(\omega_{S1},\omega_{S2})E_{1}E_{2}$, $\Phi(\Delta kL/2)=\sin c(\Delta kL/2)*e^{i\Delta kL/2}$ and $\Delta k=k_{1}+k_{2}-k_{S1}-k_{S2}$.

**Figure** S3 presents simulation results in Equation (S10) based on experimental parameters from Figure 2 in the main part, showing excellent agreement with experimental observations. Precursors are also observed between $E_{S1}$ and $E_{S2}$, generated at the rising edge of the simulated results. Figure S3a-c illustrates that the intensity ratio of the precursor to the main wave increases with larger detuning. The spikelike oscillatory structure at the leading edge results from the interference between the precursor and main waveform.^[9]^ Conversely, when the pump power is weak, the imaginary part of $\Delta k$ must be considered, resulting in $\Delta\omega_{tr1}\boldsymbol{\ll}\Delta\omega_{g1}$ under strong optical loss conditions.^[6]^ Theoretical simulations closely match the experimental results in Figure 2d-f, confirming the accuracy of the model.

**Figure S3.** a)-f) Simulation results correspond to Figure 2a-f in the main part, respectively, with parameters $\Gamma_{31}$=$\Gamma_{41}$=2π*3 MHz and $\Gamma_{21}$=$\Gamma_{31}$*0.01.

The nonclassicality of biphoton correlation can be verified by observing the violation of the well-known Cauchy-Schwarz inequality, which is defined by

$R_{2}=\frac{\left[ g^{\left( 2 \right)}(\tau) \right]^{2}}{\left[ g_{S1}^{\left( 2 \right)}(0) \right]\left[ g_{S2}^{\left( 2 \right)}(0) \right]}\leq1$ (S15)

Here, $g^{\left( 2 \right)}(\tau)$ is the normalized second-order correlation function with respect to the accidental background. $g_{S1}^{\left( 2 \right)}(0)$ and $g_{S2}^{\left( 2 \right)}(0)$ are the normalized autocorrelations of the emitted photons $E_{S1}$ and $E_{S2}$ measured by a fiber beam splitter. In our experiment, the nonzero background floor such as in Figure 2 is a result of the accidental coincidences between uncorrelated single photons. According to the measured data, we estimate that the maximum values of $g_{S1}^{\left( 2 \right)}$ and $g_{S2}^{\left( 2 \right)}$ are respectively to be 1.6 and 2.

**2. Theory of Time-Energy-Entangled W Triphoton Generation in an Atomic Vapor**

To calculate the resultant three-photon state stemming from the spontaneous six-wave mixing process (SSWM) process at the output surface of the medium, we shall work in the Schrödinger picture and commence with the following effective interaction Hamiltonian:

$H_{I}=\varepsilon_{0}\int_{V} d^{3}r\chi^{(5)}E_{1}^{(+)}E_{2}^{(+)}E_{3}^{(+)}E_{S3}^{(-)}E_{S2}^{(-)}E_{S1}^{(-)}+H.c$ (S16)

where $\chi^{(5)}$ is the fifth-order nonlinear susceptibility associated with the SSWM process, $H.c$ is the Hermitian conjugate, $E_{i}^{(+)}$represents the positive-frequency part of the input beams. Here, the generated $E_{S1}$, $E_{S2}$ and $E_{S3}$ photons are described by the quantized electric fields,

$E_{Sj}^{(-)}=\sum_{k_{Sj}} E_{Sj}a_{j}e^{i\left( k_{Sj}z-\omega_{Sj}t \right)}$ (S17)

where $a_{j}$ symbolizes the annihilation operator for the mode with the wavenumber $k_{Sj}$ and angular frequency $\omega_{Sj}$. Additionally, $E_{Sj}=i\sqrt{\hbar\omega_{Sj}/2\varepsilon_{0}n_{Sj}^{2}L}$. On the other hand, three input continuous-wave (cw) lasers $E_{1}$, $E_{2}$ and $E_{3}$are taken as classical plane waves,

$E_{1}^{(+)}=E_{1}e^{i\left( k_{1}z-\omega_{1}t \right)}$, $E_{2}^{(+)}=E_{2}e^{i\left( {-k}_{2}z-\omega_{2}t \right)}$, and $E_{3}^{(+)}=E_{3}e^{i\left( k_{3}z-\omega_{3}t \right)}$. (S18)

Substituting the electric fields of input and output into Equation (S16) we can get:

${\overset{\wedge}{H}}_{I}=W_{1}\iiint d\omega_{S1}d\omega_{S2}d\omega_{S3}\kappa\Phi(\Delta kL/2)\hat{a}_{S1}^{\dagger}\hat{a}_{S2}^{\dagger}\hat{a}_{S3}^{\dagger}e^{-i\Delta\omega t}+H.c.$ (S19)

where $\kappa\left( \omega_{i} \right)=-i\sqrt{{\varpi_{S1}\varpi_{S2}\varpi_{S3}}/{c^{3}}}\chi^{\left( 5 \right)}\left( \omega_{S1},\omega_{S2},\omega_{S3} \right)E_{1}E_{2}E_{3}$ is the nonlinear parametric coupling coefficient; $\hat{a}_{S1}^{\dagger}$, $\hat{a}_{S2}^{\dagger}$, and $\hat{a}_{S3}^{\dagger}$ represent the photon generation operators of the output modes $E_{S1}$, $E_{S2}$ and $E_{S3}$, respectively. $\Phi\left( {\Delta kL}/2 \right)=\sin c\left( {\Delta kL}/2 \right)*e^{{i\Delta kL}/2}$ is the longitudinal detuning function that determines the natural spectral width; $W_{1}$ is a constant; *L* is the length of the rubidium atomic vapor cell; $\Delta k=k_{1}+k_{2}+k_{3}-k_{S1}-k_{S2}-k_{S3}$ is the phase mismatching.

**Figure S4.** a) Energy-level diagram of hot ^85^Rb atoms for triphoton generation. The light-atom interaction occurs in a four-level triple-Λ-type atomic configuration, where the two ground states are denoted by $\left. \left| 1 \right. \right\rangle$ and $\left. \left| 2 \right. \right\rangle$, and the two excited states by $\left. \left| 3 \right. \right\rangle$ and $\left. 4 \right\rangle$. Initially, all atomic population is prepared at $\left. \left| 1 \right. \right\rangle$. To ensure no residual atomic population is distributed in $|\left. 2 \right\rangle$, an additional optical pumping beam $E_{OP}$ is applied resonantly with the atomic transition $\left. |2 \right\rangle⟷\left. |3 \right\rangle$. A weak pump laser $E_{1}$ is applied to $\left. |1 \right\rangle\to\left. |3 \right\rangle$ with a large, fixed red frequency detuning $\Delta_{1}$, $E_{2}$ and $E_{3}$ applied to atomic transition $\left. |2 \right\rangle\to\left. |4 \right\rangle$ with frequency detuning $\Delta_{2}$ and $\Delta_{3}$. The group delay for b) $E_{S2}$ and c) $E_{S3}$ as function ($L/{v_{g}}\boldsymbol{-}L/c$) of detuning $\Delta\omega_{2}$ and $\Delta\omega_{3}$, with $\Gamma_{31}$=2π*3 MHz and $\Gamma_{21}$=$\Gamma_{31}$*0.01, respectively.

The state vector of the triphotons in **Figure** S4 can then be derived from first-order perturbation theory,^[1-2]^ which is

$\left| \psi\right\rangle=\frac{-i}{\hbar}\int_{-\infty}^{+\infty} dt\overset{\wedge}{H_{I}}\left| 0 \right\rangle$ (S20)

with $|\left. 0 \right\rangle$ being the initial vacuum state. According to Reference ^[3]^ and ignoring the vacuum term that has no effect in photon clicks, the triphoton state can be formulated as:

$\left| \psi\right\rangle=\sum_{k_{S1}} \sum_{k_{S2}} \sum_{k_{S3}} F\left( k_{S1},k_{S2},k_{S3} \right)a_{k_{S1}}^{\dagger}a_{k_{S2}}^{\dagger}a_{k_{S3}}^{\dagger}\left| 0 \right\rangle$ (S21)

where the three-photon spectral function $F\left( k_{S1},k_{S2},k_{S3} \right)$ is defined as:

$F\left( k_{S1},k_{S2},k_{S3} \right)=A\chi^{(5)}\Phi\left( \Delta kL/2 \right)\delta\left( \omega_{1}+\omega_{2}+\omega_{3}-\omega_{S1}-\omega_{S2}-\omega_{S3} \right)$ (S22)

with *A* being a grouped constant. Dirac function (𝛿) comes from the time integral in the steady-state approximation, ensuring the energy conservation in the SSWM process. From the perspective of atomic population, this energy conservation implies that after a triphoton generation cycle, the population returns to its initial ground state |1⟩. $\chi^{(5)}$ is the fifth-order nonlinear susceptibility in the main part. $\Phi\left( \Delta kL/2 \right)$ is the longitudinal phase-mismatch function.

From Equation (S22), we observed that the optical responses include both linear ($\chi$) and nonlinear interactions ($\chi^{\left( 5 \right)}$) affecting the shape of the triphoton correlation wave function. Based on the energy levels depicted in Figure S4a, the perturbation chain for the generated photon in the SWM process can be expressed as:^[4, 10]^ $\rho_{11}^{(0)}\underset{\to}{\omega_{1}}\rho_{31}^{(1)}\underset{\to}{\omega_{S1}}\rho_{21}^{(2)}\underset{\to}{\omega_{2}}\rho_{41}^{(3)}\underset{\to}{\omega_{S2}}\rho_{11}^{(4)}\underset{\to}{\omega_{3}}\rho_{41}^{(5)}$. Here, $\omega_{i}$ and $\omega_{Si}$ are the frequencies of input laser and output photons, respectively. Under the dressing dressed state picture, by solving the density-matrix equations and considering a one-dimensional Doppler effect, the fifth-order nonlinear susceptibility can be written as:

$\chi^{\left( 5 \right)}\left( \delta_{2},\delta_{3} \right)=\int_{-\infty}^{\infty} \frac{1}{\varepsilon_{0}\hbar^{5}}\frac{-2f(v)N\mu_{13}\mu_{24}\mu_{23}\mu_{14}^{3}}{\left[ \left( i\Gamma_{31}+\Delta_{1} \right)d_{2}d_{3}d_{4}d_{5} \right]}$ (S23)

where $f(v)=\sqrt{\frac{m_{\mathrm{Rb}}}{2\pi k_{B}T}}e^{\frac{-m_{Rb}v^{2}}{2k_{B}T}}$ is the Maxwell-Boltzmann velocity distribution of Rb atoms in thermal motion, where $m_{\mathrm{Rb}}$ is the mass of the Rb atom, $k_{B}$ is the Boltzmann constant, $T$ is the vapor temperature, and 𝜐 is the atomic kinetic velocity; $N$ is atomic density, $\mu_{ij}$ are the electric dipole matrix elements, $\varepsilon_{0}$ is the vacuum permittivity. $d_{2}=i\Gamma_{21}+W_{D-}\delta_{2}+W_{D+}\delta_{3}$, $d_{3}=i\Gamma_{e2}+W_{D+}\delta_{2}+W_{D-}\delta_{3} +{\Delta_{2}}/2+{\Omega_{e2}}/2$, $d_{4}=i\Gamma_{e2}+W_{D-}\delta_{3}+{\Omega_{e2}}/2$, $d_{5}=i\Gamma_{e3}+W_{D-}\delta_{3} +{\Delta_{3}}/2-{\Omega_{e3}}/2$. $\Gamma_{ij}=\left( \Gamma_{i}+\Gamma_{j} \right)/2$ is the decoherence rate between $\left. |i \right\rangle$ and $\left. |j \right\rangle$; $\Delta_{i}=\omega_{i}-\omega_{j}$ is detuning defined as the difference between the resonant transition frequency $\omega_{i}$ and laser frequency $\omega_{j}$ of $E_{i}$; $\Omega_{i}={\mu_{ij}E_{i}}/\hbar$ is the Rabi frequency, *δ_i_* represents the deviations around the corresponding central frequency $\varpi_{Si}$ of generated photons that is $\omega_{Si}=\varpi_{Si}+\delta_{i}$ (*i*=1, 2, 3), where $\left| \delta_{i} \right|\ll\varpi_{Si}$. Furthermore, the frequency entanglement also can satisfy $\delta_{1}+\delta_{2}+\delta_{3}=0$. W_D-_ and W_D+_ are Doppler broadening factors, where $w_{D-}=1-v/c$, $w_{D+}=1+v/c$, respectively. When 𝑇=80 °C, the Doppler width is estimated to be approximately ∆𝐷=555 MHz, orders of magnitude larger than the Rb natural linewidth. $\Omega_{e2}$ and $\Omega_{e2}$ are the effective resonance Rabi frequencies, where $\Omega_{e2}=\left( \Delta_{2}^{2}+4\Omega_{2}^{2}+4\Gamma_{21}\Gamma_{42} \right)^{1/2}$, $\Omega_{e3}=\left( \Delta_{3}^{2}+4\Omega_{3}^{2}+4\Gamma_{21}\Gamma_{41} \right)^{1/2}$, respectively; ${\Gamma_{e2}=\left( \Gamma_{21}+\Gamma_{42} \right)}/2$ and ${\Gamma_{e3}=\left( \Gamma_{21}+\Gamma_{41} \right)}/2$ are effective resonance linewidth, which are inversely proportional to the correlation time of triphoton’s temporal correlation.

In addition to the resonance linewidths set by $\chi^{(5)}$, the triphoton temporal correlation is also influenced by the dispersion, which arises from the linear optical response. After performing calculations, we find that the linear susceptibility of the $E_{S1}$ is approximately zero ($\chi_{S1}\approx0$). Meanwhile, the linear susceptibility of the $E_{S2}$ and $E_{S3}$ fields can be represented as:

$\chi_{S2}=\int f\left( v \right)dv\frac{N\mu_{41}^{2}}{\varepsilon_{0}\hbar}\frac{-1}{W_{D+}\delta_{2}+\Delta_{2}-i\Gamma_{41}-\frac{\left| \Omega_{2} \right|^{2}}{W_{D+}\delta_{2}-i\Gamma_{10}}}$ (S24)

$\chi_{S3}=\int f\left( v \right)dv\frac{N\mu_{40}^{2}}{\varepsilon_{0}\hbar}\frac{-1}{W_{D-}\delta_{3}+\Delta_{3}-i\Gamma_{40}-\frac{\left| \Omega_{3} \right|^{2}}{W_{D-}\delta_{3}-i\Gamma_{00}}}$ (S25)

In order to discuss the optical properties of generated triphoton from a four-level system, it is essential to consider the triphoton coincidence counting measurement. Moreover, considering that the narrow bandwidths of the generated triphotons (less than GHz) are comparable to or smaller than the spectral resolution of the single-photon detectors used in our experiment. Assuming perfect detection efficiency, the averaged triphoton coincidence counting rate is defined by follow:^[11]^

$R_{cc}=\left| \left\langle0 | E_{S3}^{\left( + \right)}(\tau_{3})E_{S2}^{\left( + \right)}(\tau_{2})E_{S1}^{\left( + \right)}(\tau_{1}) | \Psi\right\rangle\right|^{2}=\left| G^{(3)} \right|^{2}$ (S26)

$G^{(3)}$ is the third-order intensity correlation function of triphoton, which can be written as:

$G^{(3)}=\left| B\left( \tau_{21},\tau_{31} \right) \right|^{2}$ (S27)

where $\tau_{21}=t_{S2}-t_{S1}$ and $\tau_{31}=t_{S3}-t_{S1}$. $t_{Si}={r_{Si}}/c$, with $r_{Si}$ representing the optical path of the photon from the producing surface of the SWM to the detector. The triphoton amplitude $B(\tau_{21},\tau_{31})$ can be expressed as:

$B\left( \tau_{21},\tau_{31} \right)=B_{0}\left( \tau_{21},\tau_{31} \right)+B_{s}\left( \tau_{21},\tau_{31} \right)$ (S28)

In Equation (S28), $B_{0}\left( \tau_{21},\tau_{31} \right)$ represents the main component of the triphoton, with its integral intervals along the $\omega_{1}$ and $\omega_{2}$ directions corresponding to the regions I shown in Figure S4b,c, respectively. These regions encompass the majority of the energy of the triphoton state, as described below:

$B_{0}\left( \tau_{21},\tau_{31} \right)=W_{21}\int_{\omega_{42}-\Omega_{c1}/2}^{\omega_{42}+\Omega_{c1}/2} \int_{\omega_{41}-\Omega_{c2}/2}^{\omega_{41}+\Omega_{c2}/2} k(\omega_{2},\omega_{3})\Phi(\Delta kL/2)e^{-i(\omega_{2}\tau_{21}+\omega_{2}\tau_{31})}d\omega_{2}d\omega_{3}$ (S29)

where *W*_21_ is a constant that absorbs all the constants and slowly varying terms. $B_{s}\left( \tau\right)$ is the Sommerfeld–Brillouin part of the triphoton. When there are several points of stationary phase, it follows that the Sommerfeld–Brillouin portion of the wave packet is defined as:^[7]^

$B_{S}\left( \tau_{21},\tau_{31} \right)=\sum_{ij} c_{ij}\frac{i\kappa\left( \omega_{i},\omega_{j} \right)\Phi(\Delta kL)}{2\pi\sqrt{\theta^{''}\left( \omega_{i} \right)\theta^{''}\left( \omega_{j} \right)}}e^{-i\left( \omega_{i}\tau+\omega_{j}\tau\right)}$ (S30)

Where $\kappa\left( \omega_{i},\omega_{j} \right)=-i\sqrt{\varpi_{S1}\varpi_{S2}\varpi_{S3}/c^{3}}\chi^{(5)}(\omega_{S1},\omega_{S2},\omega_{S3})E_{1}E_{2}E_{3}$, $\Phi(\Delta kL/2)=\sin c(\Delta kL/2)*e^{i\Delta kL/2}$.

**Figure S5.** a) Simulated triphoton coincidence counts with OD=1.6, $P_{1}$=6 mW, $\Delta_{1}$=2.0 GHz; $P_{2}$=9 mW $\Delta_{2}$=150 MHz; $P_{3}$=8 mW, $\Delta_{2}$=50 MHz; $\Gamma_{41}$=$\Gamma_{31}$=2π*3 MHz, $\Gamma_{21}$=$\Gamma_{31}$*0.01. b) Same as a), except $P_{2}$=12 mW and OD=4.6. c), d) Conditional two-photon coincidence counts corresponding to a), plotted as function of $\tau_{21}$ and $\tau_{31}$, respectively. e), f) Conditional two-photon coincidence counts corresponding to b), plotted as function of $\tau_{21}$ and $\tau_{31}$, respectively.

Triphoton coincidence counts were simulated under the experimental conditions, as shown in **Figure** S5. The results reveal a coexistence of linear and nonlinear interactions. Along the $\tau_{21}$ direction, the triphoton distribution falls within the nonlinear regime, exhibiting Rabi oscillation (Figure S5c), while along the $\tau_{31}$ direction, the distribution lies in the linear regime, exhibiting an attenuation gate function (Figure S5d). Upon increasing the power of $E_{2}$ and the optical depth (OD), a second simulation (Figure S5b) demonstrates a markedly different behavior from Figure S5a. Here, nonlinear effects dominates along the $\tau_{21}$ direction, exhibiting pronounced Rabi oscillation (Figure S5e), whereas linear interactions dominate along the $\tau_{31}$ direction, displaying a gate function shape (Figure S5f). These simulated results show improved agreement with the experimental observations.

The nonclassicality of triphoton correlation can be verified by observing the violation of the well-known Cauchy-Schwarz inequality, which is defined by

$R_{3}=\frac{\left[ g^{\left( 3 \right)}(\tau_{21},\tau_{31}) \right]^{2}}{\left[ g_{S1}^{\left( 2 \right)} \right]\left[ g_{S2}^{\left( 2 \right)} \right]\left[ g_{S3}^{\left( 2 \right)} \right]}\leq1$ (S31)

Here, $g^{\left( 3 \right)}(\tau_{21},\tau_{31})$ is the normalized third-order correlation function with respect to the accidental background. $g_{S1}^{\left( 2 \right)}(0)$, $g_{S2}^{\left( 2 \right)}(0)$ and $g_{S3}^{\left( 2 \right)}(0)$ are the normalized autocorrelations of the emitted photons $E_{S1}$, $E_{S2}$ and $E_{S3}$ measured by a fiber beam splitter. In our experiment, the nonzero background floor such as in Figure 3 is a result of the accidental coincidences between uncorrelated single photons. According to the measured data, we estimate that the maximum values of $g_{S1}^{\left( 2 \right)}$, $g_{S2}^{\left( 2 \right)}$, and $g_{S3}^{\left( 2 \right)}$ are respectively to be 1.6, 2, and 2. A clear violation of the Cauchy-Schwarz inequality is evident, with a factor of 12.6 and a normalized third-order correlation function value of 9 in **Figure 3a**, and a factor of 6.8 and a normalized third-order correlation function value of 6.6 in **Figure 3d**.

The observation of optical precursor can be precisely controlled by increasing the OD, thereby enhancing the slow-light effect. In **Figure 3d**, the the waveform of triphoton temporal correlations is observed by changing OD to 63.76 with the 120 °C temperature of the Rb vapor cell, where a distinct sharp leading edge in the waveform signifies the presence of the triphoton precursor. Here, the violation of the Cauchy–Schwarz inequality reaches a factor of 6.8 with the normalized third-order correlation function value of 6.6.

**3. Theory of Time-Energy-Entangled Conditional Two-photon Generation in an Atomic Vapor**

Compared with the biphoton and triphoton, the conditional two-photon refers to the measurement of two photons derived from the triphoton generated through SWM. The generation process remains identical to that of the triphoton, with the distinction lying in the measurement process. Moreover, considering that the narrow bandwidths of the generated triphotons (less than GHz) are comparable to or smaller than the spectral resolution of the single-photon detectors used in our experiment. Assuming perfect detection efficiency, the averaged conditional two-photon coincidence counting rate (the $E_{S3}$ photons are traced away) is defined by: ^[11]^

$R_{cc}=\left| \left\langle0 | E_{S3}^{\left( + \right)}(\tau_{3})E_{S2}^{\left( + \right)}(\tau_{2})E_{S1}^{\left( + \right)}(\tau_{1}) | \Psi\right\rangle\right|^{2}=\left| G^{(2)} \right|^{2}$ (S32)

$G^{(2)}$ is the second-order intensity correlation function of conditional two-photon, which can be written as:

$G^{(2)}=\left| B\left( \tau\right) \right|^{2}$ (S33)

where $\tau=t_{S2}-t_{S1}$. $t_{Si}={r_{Si}}/c$, with $r_{Si}$ representing the optical path of the photon from the producing surface of the SSWM to the detector. The conditional two-photon amplitude ($B\left( \tau\right)$) also comprises two components that originate from different regions in frequency space:

$B\left( \tau\right)=B_{0}\left( \tau\right)+B_{s}\left( \tau\right)$ (S34)

In the function, $B_{0}\left( \tau\right)$ is the main body of the biphoton, which can be written as:^[7]^

$B_{0}\left( \tau\right)=W_{2}\int_{\omega_{14}-\Omega_{c}/2}^{\omega_{14}+\Omega_{c}/2} \kappa\left( \omega\right)\Phi(\Delta kL/2)e^{-i\left( \omega\tau\right)}d\omega$ (S35)

$B_{s}\left( \tau\right)$ is the Sommerfeld–Brillouin precursor, which can be written as:^[7]^

$B_{S}\left( \tau\right)=\sum_{\omega_{d}} \frac{\kappa\left( \omega_{d} \right)\Phi(\Delta kL/2)}{\sqrt{-i2\pi\theta^{''}\left( \omega_{d} \right)}}e^{-i\left( \omega\tau\right)}$ (S36)

Where $\kappa\left( \omega\right)=-i\sqrt{\varpi_{S1}\varpi_{S2}\varpi_{S3}/c^{3}}\chi^{(5)}(\omega_{S1},\omega_{S2},\omega_{S3})E_{1}E_{2}E_{3}$, $\Phi(\Delta kL/2)=\sin c(\Delta kL/2)*e^{i\Delta kL/2}$ and $\Delta k=k_{1}+k_{2}+k_{3}-k_{S1}-k_{S2}-k_{S3}$.

**Figure** S6a,b present simulation results in Equation (S32) obtained under calculation conditions corresponding to the experimental parameters in D of Figure 4a,b in the main part, respectively. The simulations exhibit excellent agreement with the theoretical calculations, validating the consistency between the theoretical predictions and the numerical simulations.

**Figure S6.** a) Simulation result corresponding to Figure 4a (panel D) in the main text with parameters $\Gamma_{31}$=$\Gamma_{41}$=2π*3 MHz and $\Gamma_{21}$=$\Gamma_{31}$*0.01. b) Simulation result corresponding to Figure 4b (panel D) in the main text, with parameters $\Gamma_{31}$=$\Gamma_{41}$=2π*3 MHz and $\Gamma_{23}$=$\Gamma_{31}$*0.01.

**4 Possible SFWM processes in the 4-level system**

**Figure** S7 illustrated all possible SFWM processes based on the level structure. We have identified seven distinct SFWM processes, which are depicted in Figure S7b-h. The biphotons produced from these SFWMs processes serve as a primary source of accidental coincidences in the measured three-photon coincidence counting.

**Figure S7.** Seven possible SFWM processes in the 4-level system. a) Atomic energy-level structure for triphoton generation. b-h) Seven possible SFWM processes where the emitted biphotons may become accidental coincidences in the measured three-photon coincidence counts.^[3]^

**Figure S8.** a) Three-dimensional quantum interference formed by triphoton coincidence counts collected over 1 hour, with a time bin of 0.25ns. The power of the input $E_{1}$, $E_{2}$ and $E_{3}$ beams are 6 mW, 40 mW and 13 mW, respectively, and the corresponding frequency detuning: $\Delta_{1}$ is red detuning 2.0 GHz; $\Delta_{2}$ is red detuning 150 MHz; and $\Delta_{3}$ is red detuning 50 MHz. b) and c) Conditional two-photon coincidence counts as the function of $\tau_{21}$ and $\tau_{31}$ by tracing the third photon $E_{S2}$ and $E_{S3}$ in a), respectively.

When the power of the $E_{1}$, $E_{2}$ and $E_{3}$ set to 6 mW, 40 mW and 13 mW, respectively, a total of 2989 photon signals were recorded during a data collection period of 1 hour, including 175 unexpected counts. The triphoton coincidence counting time correlation, shown in **Figure** S8a, depicts the relative time $\tau_{21}$ ($\tau_{2}-\tau_{1}$) and $\tau_{31}$ ($\tau_{3}-\tau_{1}$) of photons. The waveform profiles of triphotons appear anti-bunching characteristics and multi-period Rabi oscillations, originating from quantum interference among multiple coherent channels in the SSWM process. Under these conditions, the triphoton correlation time exceeds 50 ns. In contrast, the biphoton and triphoton correlations in the main experiment are shorter than 10 ns due to operation in a weak linear-response regime optimized for observing optical precursor waves. Previous studies by Li et al. have explored the transition of biphoton correlation times from short to long,^[12]^ and have also investigated the long correlation times of triphotons.^[3]^ Conditional two-photon time waveforms, with $\tau_{21}$ ($E_{S1}$ and $E_{S2}$) and $\tau_{31}$ ($E_{S1}$ and $E_{S3}$) as variables, are presented in Figure S8b,c, respectively.

**5. Procedure for Reconstructing Triphoton Coincidence Counts**

In order to detect the entangled triphoton, we employed a Time-to-Digital Conversion (TDC) technique,^[3]^ which is illustrated in Figure S9a. As depicted in **Figure** S9, we establish a detection system based on two-photon coincidence circuits. Specifically, within a predetermined three-photon correlation time window, we reconstruct three individual single-photon trigger events from SPCM1, SPCM2, and SPCM3. This reconstruction is achieved through the simultaneous detection of two pairs of two-photon coincidence counts, namely ($E_{S1}$, $E_{S2}$) and ($E_{S1}$, $E_{S3}$), facilitated by an additional diagnostic SPCMD. Note that in our coincidence counting detection system, the diagnostic photon is used not for post-selection, but rather to exclude accidentals from dual pairs, uncorrelated singles, and dark counts of single-photon detectors.

In our experiment, the signal from SPCM1 ($E_{S1}$) serves as the trigger signal and is divided into two branches. One branch undergoes a delay time of 150 ns using an electric delay line. Then the two branches rejoin at the start port of TDC. The signal from SPCM3 is also delayed by 150 ns. Meanwhile, signals from SPCM2 ($E_{S2}$) and SPCM3 ($E_{S3}$) are utilized as the stop triggers; they are routed alternatively to determine whether the pulse originates from $E_{S2}$ or $E_{S3}$ during the TDC counting, with both signals connected to the stop port of the TDC. By disabling SPCM3 and removing the delay component of $E_{S1}$, we can obtain conditional two-photon counts. Additionally, we can generate biphotons by switching off LD3 and setting experimental parameters in the FWM process.

To further guarantee the detected photons that are really from SSWM, we performed one additional detection of the two-photon coincidences $E_{S3}$ and $E_{\mathrm{Diagnoise}}$ simultaneously in conjunction with the coincidences between $E_{S1}$ and $E_{S2}$ by artificially introducing the diagnosed photons $E_{\mathrm{Diagnoise}}$.^[3]^ To illustrate the functioning of each two-photon coincidence counting component, Figure S9 showcases a representative set of experimental data collected over a span of 1 hour, employing a time bin width of 0.25 ns for each SPCM. The number of detected triphotons is significantly lower than that of conditional two-photons due to the narrow time windows ($E_{S1}$, $E_{S2}$) and ($E_{S1}$, $E_{S3}$) used in the triphoton reconstruction process. These strict timing criteria are designed to ensure high purity and efficiency in triphoton selection, at the cost of filtering out a substantial portion of biphotons and triphotons. Although the recorded two-photon signals originate from the six-wave mixing process, a small number of unintended photons still arise from four-wave mixing, which acts as a subsystem within the SSWM process.

**Figure S9.** Home-made three-photon detection system and coincidence counting reconstruction. a) Sketch of the home-made triphoton coincidence detection system. b) Reconstruction of three-photon coincidence counting based on the recorded two-photon coincidence counts shown in c) and d), where c) and d) represent the recorded two-photon coincidence counts by SPCM1 and SPCM2, SPCM1 and SPCM3, respectively. d) The recorded two-photon coincidence counts between SPCM3 and SPCMD. These trigger events are plotted against the relative time differences ($\tau_{21}$, $\tau_{31}$ and $\tau_{D3}$) between clicks of the two respective single-photon detectors. The experimental data was accumulated over 1 hour with a time bin width of 0.25 ns for each SPDCM. Additional system parameters are set as follows: $P_{1}$ is 4 mW, $P_{2}$ is 40 mW, $P_{3}$ is 15 mW, $\Delta_{1}$ is red detuning 2.0 GHz, $\Delta_{2}$ is red detuning 150 MHz and $\Delta_{3}$ is blue detuning 50 MHz.

**Reference**

[1] J. Wen, S. Du, M. H. Rubin, Biphoton generation in a two-level atomic ensemble, Phys. Rev. A **2007**, *75*, 033809.

[2] J. Wen, S. Du, Y. Zhang, M. Xiao, M. H. Rubin, Nonclassical light generation via a four-level inverted-Y system, Phys. Rev. A **2008**, *77*, 033816.

[3] K. Li, J. Wen, Y. Cai, S. V. Ghamsari, C. Li, F. Li, Z. Zhang, Y. Zhang, M. Xiao, Direct generation of time-energy-entangled W triphotons in atomic vapor, Sci. Adv. **2024**, *10*, eado3199.

[4] Y. P. Zhang, A. W. Brown, M. Xiao, Opening four-wave mixing and six-wave mixing channels via dual electromagnetically induced transparency windows, Phys. Rev. Lett. **2007**, *99*, 123603.

[5] Y. P. Zhang, Z. G. Wang, Z. Q. Nie, C. B. Li, H. X. Chen, K. Q. Lu, M. Xiao, Four-Wave Mixing Dipole Soliton in Laser-Induced Atomic Gratings, Phys. Rev. Lett. **2011**, *106*, 093904

[6] S. Du, J. Wen, M. H. Rubin, Narrowband biphoton generation near atomic resonance, J. Opt. Soc. Am. B **2008**, *25*, C98.

[7] S. W. Du, C. Belthangady, P. Kolchin, G. Y. Yin, S. E. Harris, Observation of optical precursors at the biphoton level, Opt. Lett. **2008**, *33*, 2149.

[8] S. C. Zhang, J. F. Chen, C. Liu, M. M. T. Loy, G. K. L. Wong, S. W. Du, Optical Precursor of a Single Photon, Phys. Rev. Lett. **2011**, *106*, 243602.

[9] S. Du, P. Kolchin, C. Belthangady, G. Y. Yin, S. E. Harris, Subnatural Linewidth Biphotons with Controllable Temporal Length, Phys. Rev. Lett. **2008**, *100*, 183603.

[10] Z. Z. Qin, L. M. Cao, H. L. Wang, A. M. Marino, W. P. Zhang, J. T. Jing, Experimental Generation of Multiple Quantum Correlated Beams from Hot Rubidium Vapor, Phys. Rev. Lett. **2014**, *113*, 023602.

[11] Y. Li, Y. Feng, W. Li, K. Li, Y. Liu, Y. Lan, Y. Zhang, Double-dressing tri and quad-photon correlations in spontaneous six and eight-wave mixing, Phys. Scr. **2019**, *94*, 105802.

[12] K. Li, Y. Zhao, Y. Qin, Z. Chen, Y. Cai, Y. Zhang, Shaping Temporal Correlation of Biphotons in a Hot Atomic Ensemble, Adv Photon Res **2021**, *2*, 2100073.
